# Dentistry: A Multidisciplinary Approach

**DOI:** 10.3390/medicina60030401

**Published:** 2024-02-26

**Authors:** Giuseppe Minervini

**Affiliations:** 1 Saveetha Dental College and Hospitals, Saveetha Institute of Medical and Technical Sciences (SIMATS), Saveetha University, Chennai 602105, Tamil Nadu, India; giuseppe.minervini@unicampania.it; 2 Multidisciplinary Department of Medical-Surgical and Dental Specialties, University of Campania, Luigi Vanvitelli, 80138 Naples, Italy

In this special issue of Medicina, we delve into the dynamic and ever-evolving world of dentistry, highlighting the remarkable innovations that are shaping the future of oral health and clinical dentistry practice. The articles featured in this issue underscore a critical shift in the dental field: the movement towards a multidisciplinary, technology-driven approach that touches upon various branches, including restorative dentistry, prosthodontics, oral surgery, implantology, pediatric dentistry, orthodontics, and the management of temporomandibular disorders.

The fusion of traditional dental practices with cutting-edge technology is not merely a trend; it is a paradigm shift in how we approach oral health. The advent of new biomaterials, digital modeling, and advanced surgical techniques has revolutionized the way dental professionals diagnose, treat, and manage dental and oral conditions [1]. The articles within this issue also emphasize the growing importance of personalized dental care. Advancements in biomaterials and surgical techniques allow for treatments that are tailored to the unique needs and conditions of each patient. This individualized approach is crucial, especially in complex cases where a standardized treatment may not suffice [2]. Furthermore, the exploration of new frontiers, such as the use of ozonated water for the treatment of Candida infections, represents a significant step towards finding more effective, safer, and less invasive treatment options. This not only enhances patient comfort and recovery but also opens up new avenues for treating a range of dental diseases [3].

Importantly, this issue highlights the critical role of ongoing research and education in the field of dentistry. The advancements we witness today are the results of relentless inquiry and learning [4]. As such, it is imperative for dental professionals to continue engaging in lifelong learning and to remain abreast of the latest developments in their field. This commitment to education and research is what will continue to drive the field forward, ensuring that dental care remains at the forefront of medical science and technology [5,6].

In summary, “Dentistry: A Multidisciplinary Approach” offers a comprehensive overview of the current state and future potential of dental care. It exemplifies how embracing a multidisciplinary, technologically advanced, and patient-centric approach can lead to significant advancements in all branches of dentistry. As we continue to expand the boundaries of what is possible in dental care, it is these principles that will guide us towards a future where oral health is integral to overall health and well-being. These changes have been seen especially in the fields of digital dentistry, tele-dentistry, and TMD treatment [7,8,9,10]. In the field of dentistry, recent advancements emphasize a holistic, interdisciplinary approach to treatment, particularly in complex cases. Studies have shown the efficacy of techniques like diagnostic mock-ups for crown lengthening, finite element analysis for bone stress assessment, and innovative approaches for treating malocclusions and cleft lip and palate.

The article “Soft Tissue Grafting Procedures before Restorations in the Esthetic Zone: A Minimally Invasive Interdisciplinary Case Report” presents a case study of a 32-year-old male patient with esthetic concerns regarding his anterior teeth. The patient exhibited generalized clinical attachment loss, gingival recessions, and cervical non-carious lesions. The treatment plan involved plastic mucogingival surgery using tunneling connective tissue grafts and anterior ceramic laminate veneers. The surgical approach focused on improving root coverage and gingival architecture, while the restorative phase aimed to enhance dental esthetics with veneers. The case highlights the importance of an interdisciplinary approach, combining periodontal and restorative treatments, to achieve satisfying esthetic outcomes in complex cases [11]. “The Stability Guided Multidisciplinary Treatment of Skeletal Class III Malocclusion Involving Impacted Canines and Thin Periodontal Biotype” presents a case study of a 16-year-old female patient with dental and skeletal Class III malocclusion, bilaterally impacted maxillary canines, and a thin gingival biotype. The treatment involved orthognathic surgery, subepithelial connective tissue graft surgery, and a segmental arch technique. The study emphasizes the importance of a multidisciplinary approach to addressing complex dental and skeletal issues, highlighting the role of periodontal management in ensuring long-term stability and aesthetic success [12]. The article “Evaluation of the Sensitivity of Selected Candida Strains to Ozonated Water—An In Vitro Study” investigates the sensitivity of Candida strains to ozonated water. The study evaluated the impact of ozonated water at varying concentrations and exposure times on *Candida albicans*, *Candida glabrata*, and *Candida krusei strains*. The findings indicated that all the strains were sensitive to ozonated water, with increased sensitivity correlating with higher concentrations and longer exposure times. The effectiveness of ozonated water against these Candida strains was comparable to 0.2% chlorhexidine gluconate, suggesting its potential as an effective alternative for oral candidiasis treatment [3]. “Diagnostic Mock-Up as a Surgical Reduction Guide for Crown Lengthening: Technique Description and Case Report” discusses a technique using a diagnostic mock-up as a guide for crown-lengthening surgery to improve gingival architecture. This method was applied to a 30-year-old female patient concerned about her “gummy smile” and short clinical crowns. The process involved a diagnostic wax-up, a provisional overlay for surgical guidance, and final restorations with ceramic crowns and veneers. The study highlights the advantages of this technique in achieving desired aesthetic outcomes in complex dental cases [13]. The article “Injectable Resin Technique as a Restorative Alternative in a Cleft Lip and Palate Patient: A Case Report” details the treatment of a 21-year-old female patient with a unilateral left cleft lip and palate. It focuses on the use of an injectable composite resin technique for dental re-anatomization, offering a minimally invasive, efficient, and aesthetically pleasing option. This technique allowed for the successful restoration of the patient’s teeth, improving her dental anatomy and aesthetics, with positive results observed after one year [14]. The article “Neural Basis of Etiopathogenesis and Treatment of Cervicogenic Orofacial Pain” discusses the neuroanatomical and neurophysiological basis of cervicogenic pain in cervico-cranial pain syndromes, with a focus on cervico-orofacial syndromes. It covers a wide range of topics, including the clinical anatomy of the cervico-cranial junction, the role of the temporomandibular joint, and the integrative function of the cervico-cranial complex. The article emphasizes the importance of understanding neuroanatomical and neurophysiological neuromuscular relations for effective therapeutic approaches, which are primarily based on orthopedic manual and dental occlusal treatment [15]. The article “Cortical and Trabecular Bone Stress Assessment during Periodontal Breakdown–A Comparative Finite Element Analysis of Multiple Failure Criteria” by Radu Andrei Moga et al. [16] presents a numerical analysis exploring the biomechanical behavior of the mandibular bone under orthodontic forces during periodontal breakdown. It evaluates the appropriateness of various failure criteria (Von Mises, Tresca, maximum/minimum principal stresses, and hydrostatic pressure) for studying bone under these conditions. The study involves 405 simulations across 81 mandibular models with varying levels of bone loss and orthodontic movements (intrusion, extrusion, tipping, rotation, and translation). The results show that Tresca and Von Mises criteria are most suitable for bone stress analysis, displaying a coherent pattern of increasing stress across all movements and levels of periodontal breakdown. The study concludes that Tresca is better suited as a unified criterion for the study of teeth and surrounding periodontium [16]. The article “Modular Digital and 3D-Printed Dental Models with Applicability in Dental Education” explores the impact of digitalization in dental education. It discusses the development and use of modular digital dental models and 3D-printed models in teaching. The study assesses the opinions of dental students regarding these methods, emphasizing their benefits in enhancing practical skills and the understanding of dental procedures. This reflects a significant shift towards integrating advanced technology in dental education, aiming to improve student learning experiences and outcomes [17]. The article “Full-Mouth Rehabilitation of a Patient with Gummy Smile—Multidisciplinary Approach: Case Report” in Medicina describes the comprehensive treatment of a 48-year-old female patient with aesthetic concerns and disturbed masticatory function due to missing posterior teeth and a gummy smile. The treatment plan involved advanced techniques such as diode laser and piezo-surgery, implant installation, and the use of zirconia ceramic for final restorations. This multidisciplinary approach, spanning over two years, significantly improved the patient’s dental function and aesthetics. This case underscores the importance of personalized, multifaceted treatment strategies in complex dental cases [18]. The article “Evaluation of Clinical and Oral Findings in Patients with Epidermolysis Bullosa” in Medicina focuses on the oral and dental manifestations in patients with Epidermolysis Bullosa (EB), a genetic skin disorder. The study involves an assessment of clinical and oral findings in 26 EB patients, highlighting various complications like dental caries, enamel hypoplasia, and oral lesions. It underscores the unique dental care requirements of EB patients and suggests the need for specialized treatment approaches [19]. The article “A Comparative Analysis of Dental Measurements in Physical and Digital Orthodontic Case Study Models” by Elena-Raluca Baciu et al. [20] compares manual and digital orthodontic measurements on both physical and digital models. The study aims to determine the reliability of digital models in orthodontic analyses, focusing on the reproducibility of dental arch characteristics. It involves a detailed comparison of different measurement techniques applied to various types of models, including physical models created through traditional pouring and additive manufacturing as well as digital models obtained through scanning. The research concludes that both traditional and digital models are effective for orthodontic teaching, with no significant differences in the measurement results [20]. The article “The Impact of Simulated Bruxism Forces and Surface Aging Treatments on Two Dental Nano-Biocomposites—A Radiographic and Tomographic Analysis” by Amelia Anita Boitor et al. [21] investigates the effects of simulated bruxism forces and aging treatments on two dental nano-biocomposites. It focuses on the radiographic and tomographic analysis of these materials under stress. The study simulates real-life conditions like the consumption of acidic beverages and the use of at-home dental bleaching, aiming to assess the mechanical and functional behavior of these composites under such circumstances. The results provide insights into the suitability of these materials for dental restorations in patients with specific oral conditions, including bruxism [21]. The study “Different Designs of Deep Marginal Elevation and Its Influence on Fracture Resistance of Teeth with Monolith Zirconia Full-Contour Crowns” by Ali Robaian et al. [22] investigates the impact of deep marginal elevation (DME) on the fracture resistance of teeth restored with monolithic zirconia crowns. Forty premolars were divided into four groups, each undergoing different preparation and restoration procedures. The study found that fracture resistance decreased with increasing tooth structure involvement, even with monolithic zirconia crowns. However, DME up to 2 mm below the cemento-enamel junction did not negatively influence fracture resistance, suggesting its viability in clinical scenarios. The study emphasizes the importance of considering tooth preservation and material choice in restorative dentistry [22]. The article “Cranial and Odontological Methods for Sex Estimation—A Scoping Review” by Laura Maria Beschiu et al. [23] provides a comprehensive review of various methods used for sex estimation based on cranial and dental records. The study covers articles published between January 2015 and July 2022, focusing on morphometric, morphologic, and biochemical analyses in living populations, autopsy cases, and archaeological records. The review highlights that cranial and odontological sex estimation methods are highly population-specific and underscores the need for these methods to be applied to and verified in more populations. It also emphasizes the high accuracy of DNA analysis while noting the limitations and challenges of other methods for predicting sex from cranial or odontological records [23]. The article “Comparison of Mechanical Properties of Three Tissue Conditioners: An Evaluation In Vitro Study” by Marcin Mikulewicz et al. [24] compares the mechanical properties of three tissue conditioners (TC) used in dentistry. It focuses on various properties like Shore A hardness, ethanol concentration, sorption, solubility, and adhesion to denture base, evaluated under specific test conditions. The study concludes that materials containing non-phthalate plasticizers showed higher solubility and increased hardness when stored in distilled water compared to those containing phthalates. It emphasizes the importance of understanding the properties of commercial TC for optimal clinical performance and highlights the need for further research to improve these materials, especially considering the use of phthalate-free alternatives [24]. The article “Assessment and Correlation of Salivary Ca, Mg, and pH in Smokers and Non-Smokers with Generalized Chronic Periodontitis” by Saad Mohammad Alqahtani et al. [25] investigates the relationship between salivary calcium, magnesium, pH levels, and periodontitis in smokers and non-smokers. The study, conducted on 210 individuals, reveals significant differences in salivary calcium levels between smokers and non-smokers with periodontitis. It suggests that higher salivary calcium levels in smokers could be a potential marker for periodontitis progression, emphasizing the role of saliva as a diagnostic tool in periodontal diseases [25]. The article “Misfit of Implant-Supported Zirconia (Y-TZP) CAD-CAM Framework Compared to Non-Zirconia Frameworks: A Systematic Review” by Hussain D. Alsayed [26] systematically reviews studies comparing the misfit of yttria-stabilized zirconia (Y-TZP) CAD-CAM implant-supported frameworks with other materials. It includes 11 articles and covers different methods like scanning electron microscopy, one-screw tests, and 3D virtual assessment. The findings suggest that Y-TZP CAD-CAM frameworks have comparable misfits to other materials. However, due to methodological heterogeneity, the numerical misfit values are debatable, highlighting the need for standardized and well-designed in vitro and clinical studies in order to obtain definitive conclusions [26]. The article “Gaucher: A Systematic Review on Oral and Radiological Aspects” by Giuseppe Minervini et al. [27] provides a systematic review of Gaucher disease, particularly focusing on its oral and radiological manifestations. It evaluates the principal findings in the jaw using cone-beam computed tomography and X-ray orthopantomography. The study underlines the importance of dental professionals in the early diagnosis and management of Gaucher disease, emphasizing the role of dental radiographs in detecting jawbone involvement, a common feature in Gaucher patients [27]. The article “Identification of the Remains of an Adult Using DNA from Their Deciduous Teeth as a Reference Sample” by María-de-Lourdes Chávez-Briones et al. [28] presents a unique forensic case. It details the identification of an adult’s remains using DNA from their deciduous teeth, kept by the mother. This innovative approach proved crucial in this case, emphasizing the potential of using personal artifacts or saved biological samples as reference DNA in forensic investigations, especially in scenarios where conventional methods are insufficient [28]. The article “The Effect of Dentine Desensitizing Agents on the Retention of Cemented Fixed Dental Prostheses: A Systematic Review” by Mohammed E. Sayed [29] examines the impact of dentine desensitizing agents on the retention of cemented fixed dental prostheses. This systematic review compiles and analyzes data from various studies to determine how these agents affect retention. It evaluates multiple types of desensitizing agents and their interactions with different luting cements. The findings are crucial for clinical decision-making, offering guidance on selecting appropriate desensitizing agents to ensure the optimal retention of dental prostheses [29]. The article “The Impact of Anemia-Related Early Childhood Caries on Parents’ and Children’s Quality of Life” by Dila Özyılkan et al. [30] explores the relationship between anemia-related dental caries in children and their quality of life, as well as that of their parents. Utilizing the Early Childhood Oral Health Impact Scale (ECOHIS) and the Parental-Caregivers Perceptions Questionnaire (P-CPQ), the study assesses the impact of these dental issues on children and parents. The findings highlight the significant negative impact of anemia-related dental caries on quality of life, underscoring the importance of prioritizing preventive measures and timely dental treatments for affected children [30].
